# METTL7B Is Required for Cancer Cell Proliferation and Tumorigenesis in Non-Small Cell Lung Cancer

**DOI:** 10.3389/fphar.2020.00178

**Published:** 2020-02-28

**Authors:** Dongcheng Liu, Wei Li, Fuhua Zhong, Jianhua Yin, Wei Zhou, Shixuan Li, Xuefeng Sun, Jing Xu, Guofeng Li, Yuxin Wen, Jiaqing Wang, Malin Hong, Zhiqiang Cheng, Jimin Yuan, Lingyun Dai, Jichao Sun, Jigang Wang, Chen Qiu, Guangsuo Wang, Chang Zou

**Affiliations:** ^1^ Department of Clinical Medical Research Center, The Second Clinical Medical College of Jinan University, the First Affiliated Hospital of Southern University of Science and Technology, Shenzhen People’s Hospital, Shenzhen, China; ^2^ Integrated Chinese and Western Medicine Postdoctoral Research Station, Jinan University, Guangzhou, China; ^3^ Shenzhen Public Service Platform on Tumor Precision Medicine and Molecular Diagnosis, the Second Clinical Medical College of Jinan University, Shenzhen People’s Hospital, Shenzhen, China; ^4^ Laboratory of Molecular Pharmacology, Department of Pharmacology, School of Pharmacy, Southwest Medical University, Luzhou, China; ^5^ Department of Thoracic Surgery, the First Affiliated Hospital of Southern University of Sciences and Technology, Shenzhen People’s Hospital, Shenzhen, China; ^6^ Department of Pathology, the First Affiliated Hospital of Southern University of Sciences and Technology, Shenzhen People’s Hospital, Shenzhen, China; ^7^ The Second Clinical Medical College of Jinan University, the First Affiliated Hospital of Southern University of Science and Technology, Shenzhen People’s Hospital, Shenzhen, China; ^8^ Department of Respiratory and Critical Medicine, Shenzhen People's Hospital, Second Clinical Medical College, Jinan University, Shenzhen, China

**Keywords:** METTL7B, non-small cell lung cancer, proliferation, tumorigenesis, cell cycle

## Abstract

Lung cancer remains a leading cause of cancer-associated mortality worldwide, however, molecular mechanisms underlying lung cancer tumorigenesis and progression remain unknown. Here, we report evidence showing that one member of the mammalian methyltransferase-like family (METTL), METTL7B, is a potential molecular target for treatment of non-small cell lung cancer (NSCLC). METTL7B expression was elevated in the majority of NSCLC comparing to normal tissues. Increased expression of METTL7B contributed to advanced stages of tumor development and poor survival in NSCLC patients. Lentivirus-mediated shRNA silencing of METTL7B suppressed proliferation and tumorigenesis of cancer cells *in vitro* and *in vivo*. Investigation on gene expression profiles of NSCLC cells revealed that abundant cell cycle related genes were downregulated in the absence of METTL7B. Pathway enrichment analysis indicated that METTL7B participated in cell cycle regulation. Notably, CCND1, a key regulator for G1/S transition, was significantly decreased with the depletion of METTL7B, resulting in G0/G1 arrest, indicating that METTL7B is critical for cell cycle progression. Taken together, our findings implicate that METTL7B is essential for NSCLC development and progression. METTL7B might serve as a potential therapeutic target for NSCLC.

## Highlights

1. METTL7B is associated with tumorigenesis and progression in NSCLC;
2. METTL7B significantly influenced tumor growth *in vivo* and *in vitro*;
3. METTL7B serves as a potential therapeutic target for NSCLC;
4. METTL7B contributes to tumor growth through regulation of cell cycle progression.

## Background

Lung cancer is one of the most common malignant tumors worldwide (Siegel et al., 2019). There are approximately 1.8 millions of newly diagnosed lung cancer cases and approximately 1.4 millions of deaths caused by lung cancer each year (Naccache et al., 2018). Non-small cell lung cancer (NSCLC) is a major subtype (approximately 85%) of lung cancer with a poor 5-year survival rate less than 15% (Siegel et al., 2019). It is urgent to identify specific molecular biomarkers, especially previously unrecognized molecules that can be used to diagnose lung cancer at an early stage and inhibit the cancer progression.

Methyltransferases are a diverse family of proteins that are characterized by the presence of methyltransferase like domains and a structurally conserved S-adenosyl methionine (SAM) binding domain (Copeland, 2013; Yang and Bedford, 2013; Holoch and Moazed, 2015; Dan and Chen, 2016; Horning et al., 2016). Previous studies have demonstrated that methylation directly affects chromatin organization and modulate gene transcription without mutation to the gene itself (Jahan and Davie, 2015; Edwards et al., 2017). Furthermore, methyltransferases had been found to play a critical role in the development of genetic diseases, cancers, and metabolic diseases (Baqir and Smith, 2006; Yang and Bedford, 2013; Bayraktar and Kreutz, 2018). METTL7B is a member of mammalian methyltransferase-like (METTL) family. Studies had shown that members of METTL family participate various biological functions. For instance, METTL3, METTL16, METTL2B and METTL8 were found to be RNA methyltransferases (Pendleton et al., 2017; Xu et al., 2017) and play important roles in tumorigenesis (Deng et al., 2018). Yet, the role of other members of METTL family in cancer development remains largely unexplored.

In this study, we showed that METTL7B is involved in the regulation of cell cycle progression and is essential for NSCLC development. We suggest that METTL7B might serve as a potential therapeutic target for NSCLC.

## Methods and Materials

### Patients and Tissue Samples

Fifteen pairs of fresh lung cancer and adjacent normal tissues were obtained from the Second Clinical Medical College of Jinan University & Shenzhen People’s Hospital (Shenzhen, China). These patients were clinically and pathologically diagnosed at the Department of thoracic surgery, the Second Clinical Medical College of Jinan University & Shenzhen People's Hospital from 2016 to 2017. This study was approved by the Ethics Committee of Shenzhen People's Hospital. Written informed consents were obtained from the participants.

A tissue array containing a total of 94 pairs of lung cancer samples and matched adjacent normal tissues with follow-up data was obtained from Shanghai Outdo Biotech Co. Ltd. (Shanghai, China). Immunohistochemistry (IHC) assays were performed the tissue microarray chips according to standard protocols provided by Abcam. Briefly, sections were incubated with anti-METTL7B antibody (Abcam, Cat#ab110134,1:100 dilution) overnight at 4°C, and subsequently incubated with streptavidin-conjugated horseradish peroxidase. Sections were visualized with 3, 3-diaminobenzidine (DAB) kit. All IHC samples were assessed by two independent pathologists blinded to both the sample origins and the subject outcomes. The TMA was scanned using Scanscope XT (Aperio, Shanghai, China). The clinical features of the patients are listed in **Table 1**. For survival analyses, patient overall survivals stratified by expression of METTL7B, were presented as the Kaplan–Meier plots and tested for significance using log-rank tests. Differences were considered significant when P value was less than 0.05.

**Table 1:** Correlation between METTL7B expression and clinicopathological characteristics.

	Variables	METTL7B expression		Total	χ2	*p* value
		Low	High		
Age (year)					0.604	0.437
	⩽60	15	28	43		
	>60	14	37	51		
T stage					5.088	0.024*
	T1/T2	26	44	70		
	T3/T4	3	21	24		
Sex					0.551	0.458
	Female	11	30	41		
	Male	18	35	53		
TNM stage					3.631	0.057
	I/II	18	31	49		
	III/IV	5	25	30		
	Null			15		
N stage					1.893	0.169
	N0	15	27	42		
	N1/N2/N3	8	29	37		
	Null			15		
M					0.458	0.499
	M0	29	63	92		
	M1	0	1	1		
	Null			1		
Grade					1.879	0.170
	I/II	17	50	67		
	III	10	15	25		
	Null			2		
EGFR					4.590	0.032*
	Negative	24	42	66		
	Positive	1	13	14		
	Null			14		
ALK					1.076	0.300
	Negative	21	50	71		
	Positive	3	3	6		
	Null			17		
VEGF					0.024	0.878
	Negative	12	28	40		
	Positive	17	37	54		
VEGF					0.024	0.878
	Negative	12	28	40		
	Positive	17	37	54		
PD-L1						0.303^#^
	Negative	5	6	11		
	Positive	21	52	73		
	Null			10		
Survivin						1.000
	Negative	4	9	13		
	Positive	17	40	57		
	Null			24		

*The p value < 0.05 is regarded as statistically significant.

^#^Fisher's exact test was used.

### Cell Lines and Cultures

Lung cancer cells (A549, PC-9), 293T cells were purchased from the American Type Culture Collection (ATCC). These cells were authenticated using short tandem repeat (STR) by Genetic testing Biotechnology Corporation (Suzhou, China). A549 and PC-9 were cultured in RPMI-1640 (Hyclone, Thermo Scientific) medium supplemented with 10% fetal bovine serum (Hyclone, Thermo Scientific) at 37°C in an incubator with 5% CO_2_. 293T cell were cultured in Dulbecco's modification of Eagle's medium (Hyclone, Thermo Scientific) supplemented with 10% fetal bovine serum (Hyclone, Thermo Scientific) at 37°C in an incubator with 5% CO_2_. All of cell lines were confirmed with negative mycoplasma contamination.

### Lentivirus Virus Production and Transduction

For lentivirus mediated shRNA knockdown, pLKO.1-GFP construct together with packing plasmids psPAX2 and pMD2.G were co-transfected into 293T cells. Viruses were collected at 48 hours and 72 hours after transfection and then added to A549 or PC-9 cells with polybrene (8 μg/ml, Sigma). Forty-eight hours after infection, Puromycin was added to the culture medium for stable cell selection. The sequence targeting Human METTL7B were as follow, shMETTL7B-1: 5'-GGGAAAGGCTGTCAAATAA-3', shMETTL7B-2: CAGGGCAATCTCTAACTTCAA. Real-time quantitative PCR(RT-qPCR) and Western blot were performed to determine the knockdown efficiency.

### Cell Viability Assay

The cell viability was measured using Cell Counting Kit-8 (CCK-8) (MedChem Express, Monmouth Junction, NJ, USA) according to the manufacture's recommendations. Briefly, A549 and PC-9 cells with METTL7B shRNA or negative control shRNA were seeded in 96 well plate. Cell proliferation was documented every 24 h for 3 days. The number of viable cells was assessed by measurement of the absorbance at 450 nm using a microtiter plate reader (BIO-TEK Instruments, Winooski, VT, USA).

### Colony Formation Assay

Cells were seeded in six-well plates at a density of 10^3^ cells/well. After incubation at 37°C for 14 days, colonies were fixed with 4% paraformaldehyde and stained by crystal violet for 15 min at room temperature and photographed by a camera. Macroscopic colonies of each well were counted.

### *In Vivo* Tumorigenesis Assay

Animal study was approved by the Jinan University Institutional Animal Care and Use Committee. Experimental procedures were performed in accordance with the Guide for the Care and Use of Laboratory Animals (National Institutes of Health Publication No. 80-23) and according to the institutional ethical guidelines for animal experiments. Male BALB/c nu/nu mice (4–5 weeks old) purchased from the Laboratory Animal Center of Shanghai, Academy of Science Chinese (Shanghai, China), were housed under specific pathogen-free conditions. Mice were randomly divided into two groups with five mice in each group. Viable cells (3 × 10^6^ cells/mice) were injected subcutaneously into the flanks of mice. Ctrl group was injected with shCTRL-A549 cells; shMETTL7B group was injected with shMETTL7B-1 cells. Ten days after cell injection, the length (L) and width (W) of tumor xenografts were measured at a three-day interval with a Vernier caliper. Tumor volumes were calculated (V = W^2^ × L/2). Bioluminescent imaging was performed on tumors on day 35. The animals were sacrificed under general anesthesia with chloral hydrate (5%, 100 μl/10 g). The tumors were removed, weighted, and fixed for immunohistochemical experiments with primary antibodies: anti-METTL7B (1:100 dilution, Abcam, Cat#ab110134), anti-Ki67 (1:400 dilution, Cell signaling Technology, Cat# ab92742).

### RNA Isolation and Microarray Hybridization

Total RNA from A549 cells treated with METTL7B shRNA or control shRNA was extracted with a Qiagen RNeasy Mini Kit according to the manufacturer's instructions. RNA concentration and purity were measured with the NanoDrop 2000 (Thermo Scientific, Pittsburgh, PA). The Affymetrix PrimeView Human Gene Expression Array (Affymetrix, SantaClara, CA) was used to assess the differential mRNA expression in shCTRL and shMETTL7B cells and performed by CapitalBio Corporation (Beijing, China) according to the manufacturer's instructions. The PrimeView microarray comprises more than 36,000 transcripts mapping over 20,000 unique genes.

### Microarray Data Analysis

Affymetrix GeneChip Command Console Software was used to analyze microarray data and summarize the probe level information (Hou et al., 2015). Significance Analysis of Microarrays software was used to identify differentially expressed genes (DEGs) between vector control group and shMETTL7B group, and the criteria for DEGs were FDR <0.05 and fold change >1.5 or <0.5. The program Ingenuity Pathway Analysis (IPA, www.ingenuity.com) was used to draw functional pathways relevant to the DEGs identified. The microarray data have been submitted to the NCBI Gene Expression Omnibus (GEO accession number GSE142278).

### RNA Extraction and Real-Time Quantitative PCR Assays

Total RNA was extracted from cells using TRIZOL Reagent (Invitrogen, USA), and cDNA was synthesized from 1 μg of RNA with the M-MLV Reverse Transcriptase Kit (Promega, USA) as recommended by the manufacturer. Real-time quantitative PCR reactions for the quantification of gene expression were performed with Bio-Rad iQ5 Real Time PCR System. The primers sequences used in this study were listed in **Supplementary Table S1**.

### Western Blot

Total protein was extracted and protein concentration was determined with the BCA Protein Assay Kit (Pierce, Rockford, IL, USA). Equivalent amounts of proteins samples were uploaded and separated by SDS-PAGE and then electro-transferred to polyvinylidene difluoride (PVDF) membranes (Millipore Corp, Atlanta, GA, US). The membranes were blocked in 5% non-fat dry milk powder at room temperature for 1 h, and then incubated overnight at 4°C with primary antibodies: anti-METTL7B (1:1000 dilution, Abcam, Cat #ab110134), anti-GAPDH (1:1000 dilution, Cell Signaling Technology, Cat#5174S). Membranes were then incubated with HRP-conjugated secondary antibodies at room temperature for 1 h. The signals of bands were detected by ECL reagents.

### Cell Cycle Analysis

Cell cycle profiles were analyzed by flow cytometry with standard propidium iodide (PI) staining method (Beyotime Biotechnology, China) according to the manufacture's manual. Cells were harvested and washed once with PBS, and fixed in 70% ethanol at -20°C overnight. Cells were washed with PBS twice and resuspended in PBS containing PI at a final concentration of 10 g/ml and RNase A (20 g/ml). The samples were kept at room temperature for 30 min in the dark and then analyzed using a FACSCalibur flow cytometry (BD Biosciences). The data were analyzed using FlowJo software (Tree Star, Ashland, OR, USA).

### Statistical Analysis

All data were expressed as mean ± standard deviation (S.D). We employed SPSS 19.0 (SPSS, Chicago, IL) to conduct all the statistical analysis. Student's t-test were used to evaluate the differences between two comparison groups, One-way ANOVA was used for multiple-group comparisons, Chi-square test and Spearman's rank test were used for correlation between METTL7B expression and clinicopathological characteristics (or Fisher's exact test if appropriate). Survival analyses were plotted using Kaplan-Meier curves and compared using the log-rank test. P value less than 0.05 was regarded as statistically significant.

## Results

### METTL7B Is Up-Regulated in NSCLC Tissues

**Figure 1:**
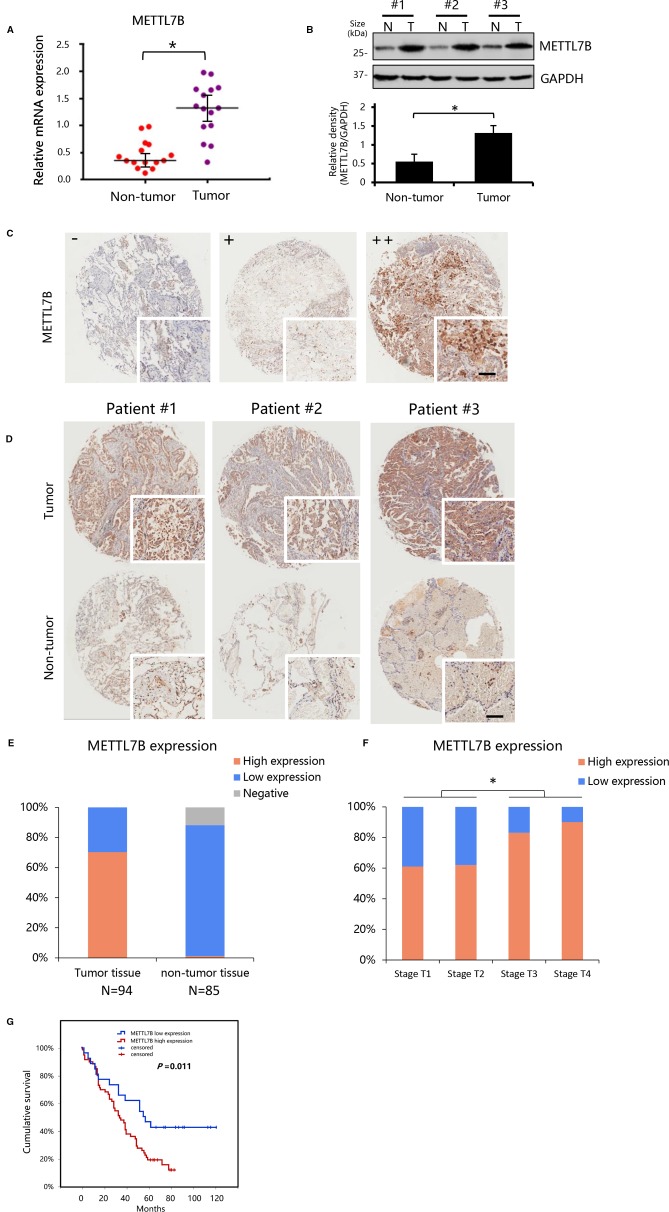
METTL7B up-regulated in clinical lung cancer tissues. **(A, B)** The expression of METTL7B evaluated in 14 pairs of clinical lung cancer tissues and their matched non-tumor tissues by qRT-PCR (p < 0.05) and Western blot (Data from three representative patients were shown). **(B)** Data in lower panel represented the density quantification of bands (normalized to GAPDH) for each experimental group, *p < 0.05 vs. non-tumor. N, non-tumor tissues; T, tumor tissue. **(C**, **D)** Immunohistochemical staining of METTL7B protein in tumor tissue and matched non-tumor tissues from non-small cell lung cancer (NSCLC) tissue microarray of 95 cases shown at low (×40) and high (×400; bar = 100 *μ*m) magnification. **(C)** Representative sections for staining intensity “-” negative, “+” low expression, “++” high expression. **(D)** Immunohistochemical staining images from three representative patients. **(E)** The METTL7B expression in lung cancer tissues and their matched non-tumor tissues groups. **(F)** METTL7B expression in tumor tissue with different Tumor (T) stages. **(G)** Kaplan-Meier survival curve analysis of NSCLC patients with high or low METTL7B expression.

To explore the role of METTL7B in NSCLC, we first measured the expression of METTL7B in a small cohort of 15 pairs of clinical lung cancer tissues and their matched adjacent non-tumor tissues by qRT-PCR and Western blot. Our results showed that the expression level of METTL7B was significantly higher in lung cancer tissues compared to matched adjacent normal lung tissues in both mRNA and protein level (**Figures 1A, B**).

To confirm our finding, we performed an NSCLC tissue microarray (TMA) analysis for a large cohort with 94 patients. METTL7B protein level were verified by IHC staining on TMAs. Positive staining of METTL7B was predominately found in the cytoplasm of tumor cells (**Figure 1C**). Tumor cells showed strong expression of METTL7B protein, while residual normal mammary epithelial cells presented low IHC staining intensity (**Figure 1D**). More importantly, we found that the nearly 70% (66/94) of NSCLC tissue overexpressed METTL7B while it expressed at a lower level in 97% (84/85) of normal tissues (**Figure 1E**), indicating that the METTL7B plays an important role in cancer development in NSCLC. Notably, a significant correlation between METTL7B expression and advanced tumor (T) status was found (*p*=0.024) (**Figure 1F**, **Table 1**), suggesting that METTL7B expression is correlated with tumor progression in NSCLC. To further explore the role of METTL7B in NSCLC, we screened the correlation of expression between METTL7B and cancer related genes, including epidermal growth factor receptor (EGFR), Anaplastic lymphoma kinase (ALK), Vascular endothelial growth factor (VEGF), Programmed cell death-1 (PD-1), and Survivin. Interestingly, we found that the expression of EGFR was associated with METTL7B (*p* = 0.032) (**Table 1**). More importantly, elevated METTL7B expression was significantly associated with shortened survival of lung cancer patient (**Figure 1G**, *p* = 0.011). Taken together, our results showed that up-regulated METTL7B is associated with tumorigenesis and poor clinical outcomes in NSCLC.

### METTL7B Is Essential for Cancer Cell Proliferation in NSCLC *In Vitro*

**Figure 2:**
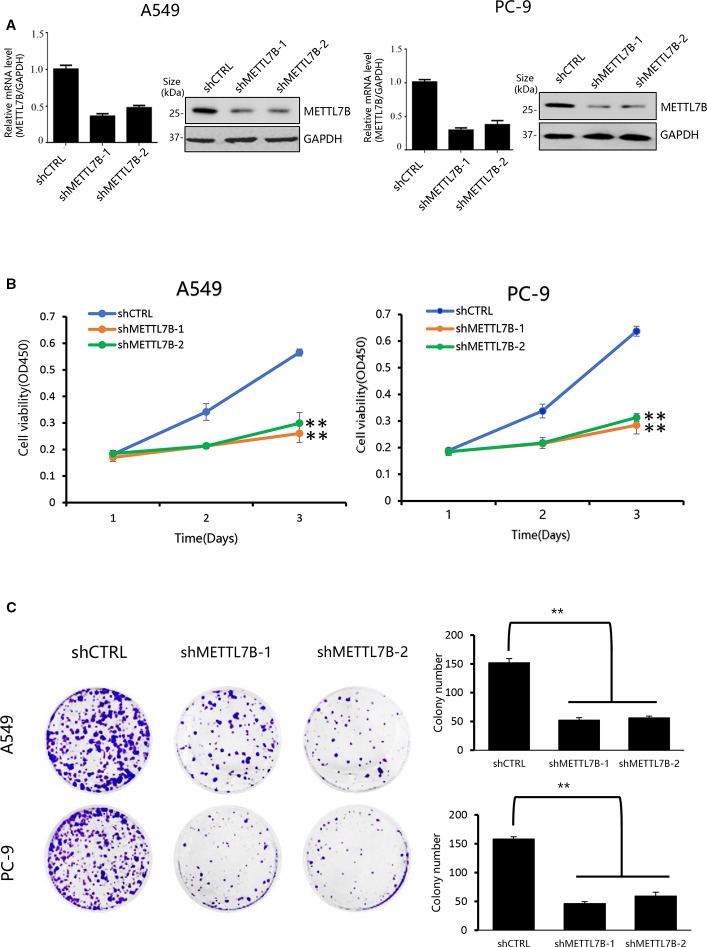
Knockdown of METTL7B inhibits the proliferation of lung cancer cells *in vitro*. **(A)** A549 and PC-9 cells transducted with sh-METTL7B (shMETTL7B-1, -2) or shControl RNA (shCTRL) mediated by lentivirus for 48 h. The knockdown efficiency was confirmed by qRT-PCR and Western blot. Error bars = mean ± SD, n = 3. **(B)** Cell viability assay. After lentivirus infection, cells were collected and seeded in 96-well plates (2,000 cells/well). CCK8 assay applied to measure the cell viability for 3 days. Data are presented as mean ± SD, n = 3. One way ANOVA, **p < 0.01, vs. shCTRL group. **(C)** Colony-formation assay performed with shMETTL7B or shCTRL cells. After lentivirus infection, cells were collected and seeded in six-well (1,000 cells/well) plate. After incubation for 14 days, colonies were stained by crystal violet and photographed. One way ANOVA, **p < 0.01, vs. shCTRL group. All values are the average of triplicate experiments with the SD indicated by the error bars.

In order to understand the biological functions of METTL7B in the progression of lung cancer, loss of function lung cancer cell model was applied by using lung adenocarcinoma cell line A549 and PC-9 since METTL7B was up-regulated significantly in adenocarcinoma tumor but not in lung squamous carcinoma based on TCGA dataset (http://ualcan.path.uab.edu/index.html) (**Supplementary Figure S1**). These cells were subjected to lentivirus mediated interference targeting METTL7B with two different shRNAs. The knockdown efficiency was examined by qPCR and Western blot (**Figure 2A**). Firstly, we performed cell proliferation assay to see the role of METTL7B in cancer cell growth. Interestingly, in the absence of METTL7B, we observed that the cell growth and proliferation were significantly inhibited in both cell lines (**Figure 2B**). To confirm these data, we then determined the viability of cells by performing crystal violet staining. Indeed, the formation and growth of colonies in A549 and PC-9 were eliminated significantly with the treatment of METTL7B shRNAs (**Figure 2C**). Therefore, our results revealed that METTL7B is essential for cell growth and proliferation of NSCLC cells *in vitro*.

### METTL7B Is Required for Lung Tumorigenesis *In Vivo*

**Figure 3:**
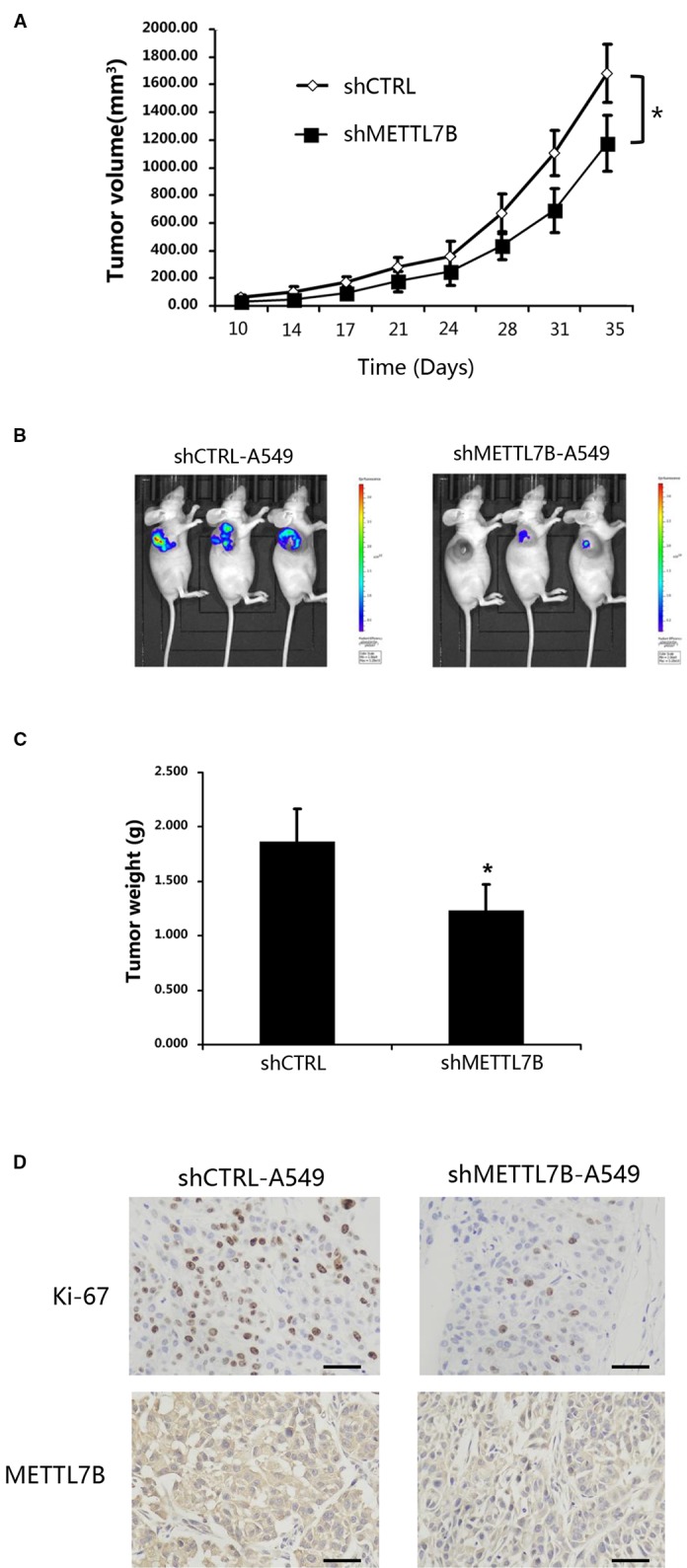
METTL7B is essential for cancer cell proliferation *in vivo.* ShMETTL7B-A549 or shCTRL-A549 cells were subcutaneously injected into BALB/c-nu mice. **(A)** Ten days after cell inoculation, tumor was measured twice a week. Tumor volumes were calculated. **(B)** Bioluminescent imaging of tumors with shMETTL7B-A549 or shCTRL-A549 cells in BALB/c-nu mice on day 35 (data from three representative mice of each group were shown). **(C)** On the 35th day, mice were sacrificed and tumor xenografts were excised and weighted. **(D)** Immunohistochemical staining with indicated antibodies. Scale bar, 50 μm. (Data represent mean ± S.D, *p < 0.05 indicates significant difference between the groups. Five mice were used for each group).

To test whether METTL7B is required for tumorigenesis *in vivo*, A549 cells stably expressing shMETTL7B or shCTRL were subcutaneously inoculated into BALB/c nude mice (five mice per group). After transplantation, tumor volume (width^2^ × length/2) was examined twice a week. To confirm the role of METTL7B in tumor growth, lung tumorigenesis was evaluated by histopathology after mice were scarified 35 days post tumor injection. The proliferation marker of Ki-67 were measured. As shown in **Figures 3A–C**, xenograft tumor growth was greatly inhibited in mice bearing shMETTL7B, with a notable decrease in tumor sizes and weights (*p* < 0.05). The lowered expression of Ki67 from IHC staining was also observed in shMETTL7B xenograft tumors (**Figure 3D**), suggesting the decreased proliferative cells in METTL7B knockdown xenograft tumors when compared with the control samples. Taken together, these findings indicated that METTL7B is also essential for lung cancer tumorigenesis *in vivo*.

### METTL7B Regulates Cell Cycle Progression

**Figure 4:**
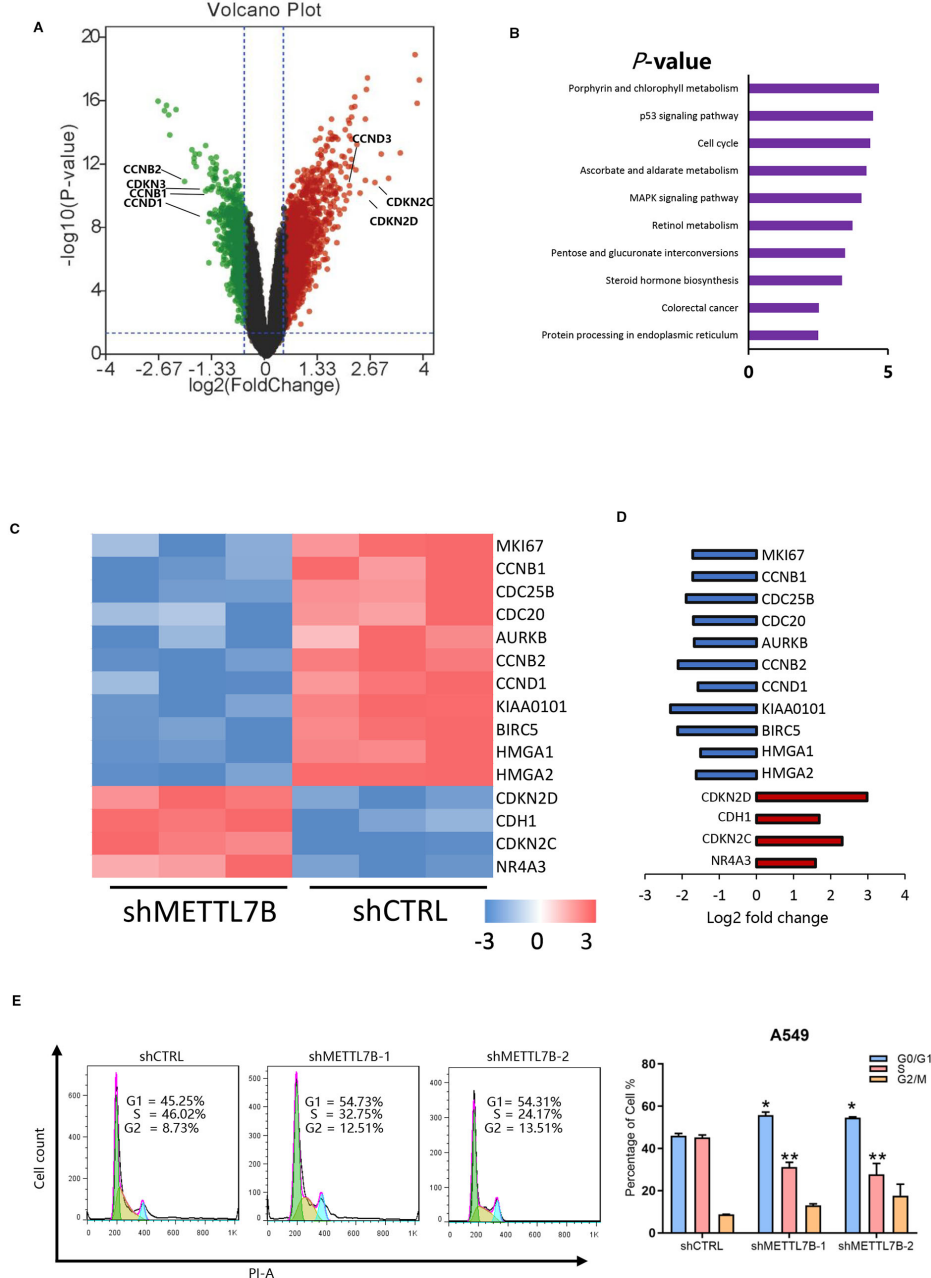
METTL7B regulates cell proliferation through modification of mRNA expression of cell cycle genes in non-small cell lung cancer (NSCLC). Affymetrix microarrays were used to analyze transcript profiles between shMETTL7B-A549 and shCTRL-A549 cells. **(A)** Volcano Plot demonstrated the distribution of the differentially expressed genes between shMETTL7B group and shCTRL group. The X-axis represents the logarithm conversion of the fold difference to base 2 and the Y-axis represents the logarithm conversion of the corrected significant levels to base 10. Values with a FC> 1.5, and *p*-value < 0.05 are indicated in red and are considered significantly up-regulated; while values with a FC<-1.5 and *p*-value < 0.0.5 are indicated in green and are considered down-regulated. The black dots represent genes with no significant differences. **(B)** Kyoto Encyclopedia of Genes and Genomes (KEGG) pathway analysis of differentially expressed mRNAs in A549 cells treated shMETTL7B. Cell cycle related genes are one of top three most significantly enriched signaling pathways with a *p*-value of 4.3E−5. **(C)** Heat map representation of microarray analysis of the cell cycle-specific differentially expressed transcripts within the shCTRL-A549 and shMETTL7B-A549 cells. **(D)** Fold change of cell cycle specific genes in (C). Up-regulated genes were in red and down-regulated genes were in blue in shMETTL7B-A549 cells. **(E)** Cell cycle analysis. (*p < 0.05, **p < 0.01). Error bars = mean ± SD, n = 3.

To explore the mechanism of METTL7B on lung cancer development, global gene expressions of shMETTL7B-A549 and shCTRL-A549 cells were evaluated using Affymetrix GeneChip PrimeView Human Gene Expression Arrays. A total of 1422 differentially expressed genes were identified with 904 genes up-regulated and 518 down-regulated (**Supplementary Table S2**). The distribution of differentially expressed genes by fold change (FC) between the shMETTL7B group and the shCTRL group (|FC| > 1.5, *p* < 0.05) was shown in a volcano graph (**Figure 4A**). Ingenuity pathway analysis (IPA) demonstrated interactions (direct/indirect) of METTL7B with genes involved in cellular signaling pathways including Cyclins and Cell Cycle Regulation(CCND1,CCNB1); p53 Signaling(BIRC5,HDAC9); TGF-β Signaling (BMP4,JUN); and NRF2-mediated Oxidative Stress Response(EIF2A,STIP1). To confirm these findings, 20 selected genes involved in these signaling pathways were further validated using qRT-PCR analysis (**Supplementary Figure S2**). Interestingly, we observed that a large number of cell cycle related genes were modified (**Figures 4A, C, D**). In addition, Kyoto Encyclopedia of Genes and Genomes (KEGG) Pathway Enrichment Analysis shown that METTL7B participated in cell cycle regulation (**Figure 4B**). Furthermore, IPA network shown that cell cycle regulator Cyclin D1(CCND1) was the centralized “hub” molecule linking multiple interacting genes (**Supplementary Figure S3**). In the absent of METTL7B, CCND1 was down-regulated while cyclin-dependent kinase 4 inhibitor (CDKN2D) was up-regulated (**Figures 4A, C**), indicating that G0/G1 transition might be inhibited. Based on these clues, we analyzed the cell cycle progression of cells with or without shMETTL7B treatment by using flow cytometry. Indeed, cells were arrested at G0/G1 phase in the absence of METTL7B (**Figure 4E**). Taken together, our results indicated that METTL7B promotes tumorigenesis by regulating cell cycle progression.

## Discussion

In this study, we demonstrated the role of METTL7B in tumor development and progression of NSCLC. Firstly, METTL7B is up-regulated in both mRNA and protein levels in lung cancer tissues, which is closely associated with advanced stages of tumor development and low survival rate in patients with NSCLC. This provides a potential for using METTL7B as a diagnostic biomarker. Secondly, growth and tumorigenesis of lung cancer cells are inhibited when METTL7B is knocked down, suggesting that METTL7B is a potential target for lung cancer therapy. Third, METTL7B might be a cell cycle regulator that mediates G1/S transition in cancer cell.

METTL7B was initially found to be a Golgi related methyltransferase (Wu et al., 2004), however, its function in cancers is rarely studied. Previous study showed that METTL7B might serve as a biomarker for diagnosis and tumor progression in papillary thyroid carcinoma (Cai et al., 2018). METTL7B enhanced migration and invasion of thyroid carcinoma cells through promote TGF-β1-induced epithelial-mesenchymal transition (EMT) (Ye et al., 2019). In our study, we provide evidence showing that METTL7B is a potential therapeutic target for NSCLC.

A number of studies have demonstrated that CCND1 is a key driver of malignant transformation and is frequently overexpressed in lung cancer which attributed to cancer cell proliferation (Fu et al., 2004; Gautschi et al., 2007; Musgrove et al., 2011). In addition, CCND1 overexpression causes a number of potentially oncogenic responses in experimental models and is associated with poor patient outcome (Musgrove et al., 2011). Here, our data shown that depletion of METTL7B caused markedly decreased of CCND1 and eventually arrested cancer cells in G0/G1 phase and inhibited cancer cell proliferation *in vitro* and *in vivo.* We did not observe significant enhancement of apoptosis in shMETTL7B treated cells (data not shown) indicating that cell cycle arrest is the main cause of proliferation inhibition in cancer cells. Thus, targeting METTL7B could provide a novel therapeutic strategy for treatment of NSCLC *via* inhibiting CCND1 and cell cycle regulation. To better understand the function of METTL7B, efforts should be made on the mechanisms of how METTL7B regulates CCND1.

Previously studies found that METTL7B can be induced by mutant P53 but not wildtype P53 protein through interaction in the upstream promoter region of METTL7B (Neilsen et al., 2011). P53 is the most frequently mutated gene in cancer and many mutant p53 proteins exert oncogenic gain-of-function (GOF) properties that promote cancer cell growth and metastasis (Senturk et al., 2014; Bykov et al., 2018), however, the underlying mechanism remains unclear. From our gene microarray data, IPA and KEGG analysis revealed that METTL7B was involved in p53 pathway. It is possible that the induction of METTL7B is one of the mechanisms to explain how mutant p53 promotes cancer cell proliferation. We plan to explore whether or not deletion of METTL7B abolishes the oncogenic function of mutant p53 proteins in future studies.

There are over 27 members in METTL family, yet, only several of them had been studied (Lin et al., 2016; Wang et al., 2016; Pendleton et al., 2017; Ignatova et al., 2019). Since limited conserved domains shared among this family (Ignatova et al., 2019), their functions had been found to be various. Recently studies had shown that some members of METTL family played important role in tumorigenesis through various mechanisms. For instance, METTL3 is essential for differentiation of leukemic cells by inducing m6A modification within the coding region of cell cycle associated mRNA transcript, eventually promotes translation of a large subset of oncogenic mRNAs (Choe et al., 2018). METTL13 recognizes eEF1A protein N terminus and methylates eEF1A and modulates mRNA translation (Jakobsson et al., 2018). To date, there is no evidence showing that METTL7B exerts methylation activity. It is worthy to explore the potential function of RNA methylation activity because: (i) METTL7B contains a S-adenosylmethionine binding site domain which shares with many methylation protein (Turro et al., 2006); and (ii) METTL7B colocalized in cytoplasm that might affects mRNA stability *via* direct RNA-protein interaction (Ignatova et al., 2019). It is possible that METTL7B could modify the methylation status of cell cycle related gene, including CCND1. We expect that the functions of METTL7B in mRNA methylation regulation will be deciphered soon.

In summary, our study illustrates that METTL7B is aberrantly overexpressed in human primary lung tumors with advanced tumor stages. Silencing METTL7B leads to G0/G1 phase arrest in cancer cell and significantly reduces the cell proliferation capabilities both *in vitro* and *in vivo*. METTL7B could be a potential therapeutic target for NSCLC treatment. Moreover, our study reveals critical signaling pathways and genes that involved in regulation of the cancer development network of METTL7B which will be a valuable resource for future clinical application.

## Data Availability Statement

The data supporting our findings can be found in the article. All of the data generated in this study are available on request.

## Ethics Statement

This study was approved by the Ethics Committee of Shenzhen People's Hospital. The patients/participants provided their written informed consent to participate in this study. Animal study was approved by the Jinan University Institutional Animal Care and Use Committee.

## Author Contributions

CZ and GW conceived and designed the research. DL, WL, FZ, JYi, WZ, SL, XS, JX, GL, YW, JiaW, MH, ZC, JYu, LD, and JS performed the experiments and data analysis. DL and WL wrote the manuscript. JiW, CQ, CZ, and GW revised the manuscript.

## Funding

The work was supported by grants from the Natural Science Foundation of Guangdong Province, China (No. 2016A030313035), the 2016 Shenzhen Overseas High-level Talents Innovation and Entrepreneurship Plan (No.KQTD2016113015442590), the National Natural Science Foundation of China (No. 81702889), the Shenzhen Public Service Platform on Tumor Precision Medicine and Molecular Diagnosis, the Shenzhen Cell Therapy Public Service Platform. The Shenzhen Economic and Information Committee “Innovation Chain and Industry Chain” integration special support plan project (20180225112449943), Shenzhen Public Service Platform on Tumor Precision Medicine and Molecular Diagnosis. The Guangdong Provincial Natural Science Foundation (2018A030313743).

## Conflict of Interest

The authors declare that the research was conducted in the absence of any commercial or financial relationships that could be construed as a potential conflict of interest.

The reviewer LF declared a past co-authorship with one of the authors CZ to the handling editor.

## Supplementary Material

Supplementary Figure S1 The expression of METTL7B in lung adenocarcinoma carcinoma and lung squamous carcinoma from TCGA dataset.

Supplementary Figure S2 Validation the genes expression of the microarray data in A549 cells treated with shMETTL7B or shCTRL by qRT-PCR.

Supplementary Figure S3 IPA network analysis of gene microarray.

Supplementary Table S1 Primers used for real-time RT-PCR.

Supplementary Table S2 Differentially expressed genes from microarray in shMETTL7B-A549 compared with shCTRL-A549 cells.
