# Supplementary figures and images for: METTL7B Is Required for Cancer Cell Proliferation and Tumorigenesis in Non-Small Cell Lung Cancer
Source: Front Pharmacol. 2020 Feb 28;11:178. doi: 10.3389/fphar.2020.00178 (PMC7059849; doi:10.3389/fphar.2020.00178)

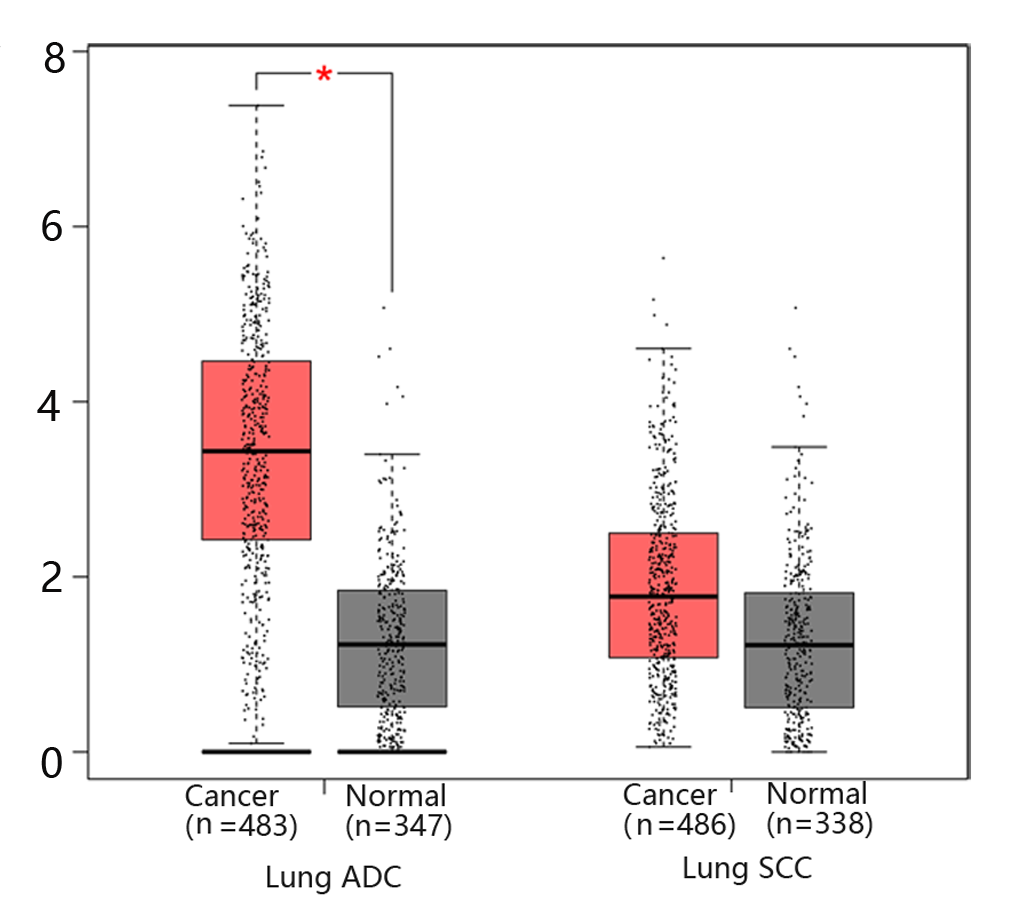

Supplement: Supplementary Figure S1 — The expression of METTL7B in lung adenocarcinoma carcinoma and lung squamous carcinoma from TCGA dataset. [file Image_1.tif]

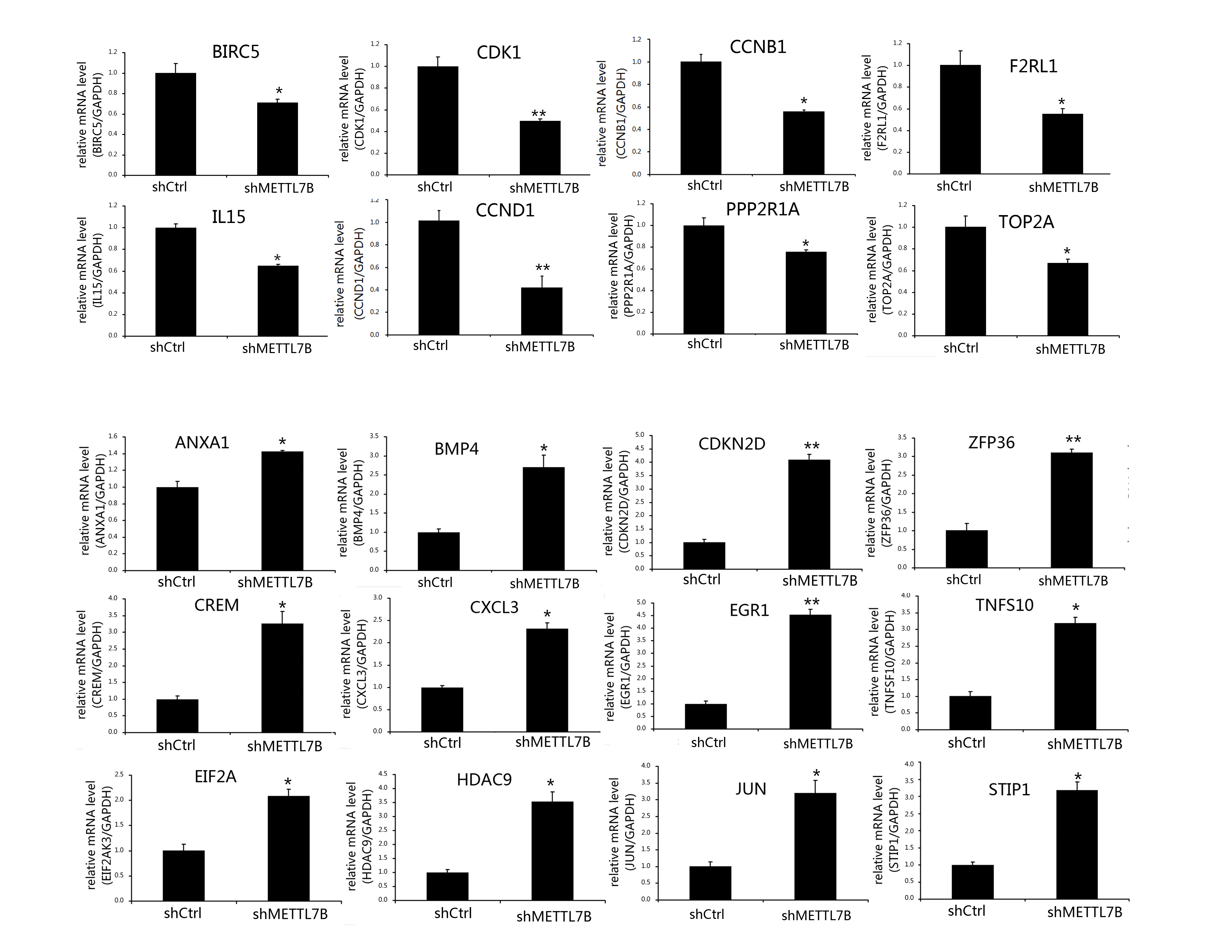

Supplement: Supplementary Figure S2 — Validation the genes expression of the microarray data in A549 cells treated with shMETTL7B or shCTRL by qRT-PCR. [file Image_2.tif]

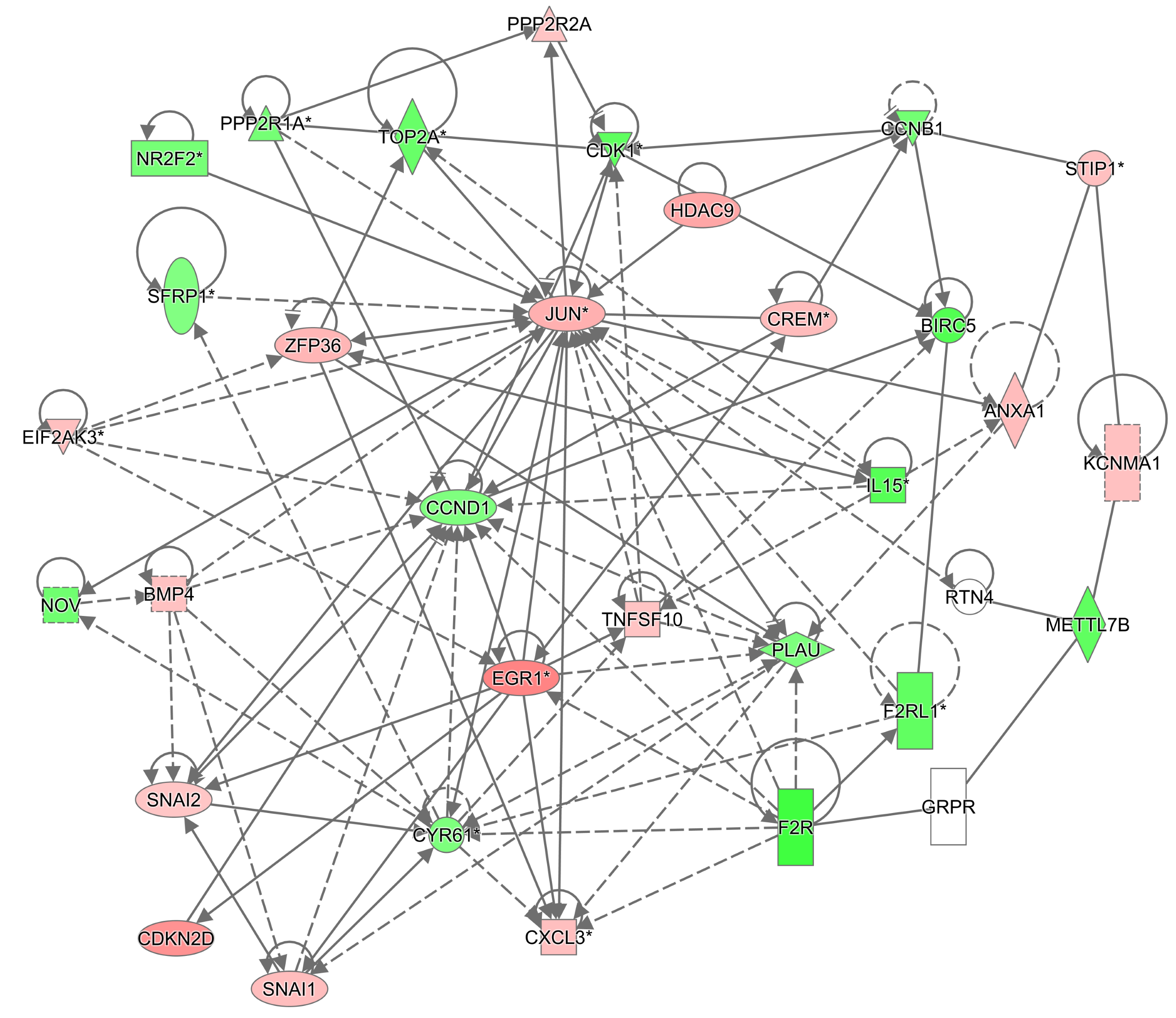

Supplement: Supplementary Figure S3 — IPA network analysis of gene microarray. [file Image_3.tif]
