# Supplementary material for: METTL7B Is Required for Cancer Cell Proliferation and Tumorigenesis in Non-Small Cell Lung Cancer
Source: Front Pharmacol. 2020 Feb 28;11:178. doi: 10.3389/fphar.2020.00178 (PMC7059849; doi:10.3389/fphar.2020.00178)
Supplement: Supplementary Table S1 — Primers used for real-time RT-PCR. [file Table_1.docx]

**Supplementary Table 1.**

**Primers used for real-time RT-PCR.**

| **Gene** | **Sequences(5’ ￫ 3’)**  **(Forward)** | **Sequences(5’ ￫ 3’)**  **(Reverse)** | **Product length (bp)** | **GenBank accession number** |
| --- | --- | --- | --- | --- |
| METTL7B | CCAGATAAAGGGGCTTACAGGAG | TCAGCCATGCTCTTTGTCAGG | 159 | NM_152637.3 |
| BIRC5 | ACCGCATCTCTACATTCAAG | CAAGTCTGGCTCGTTCTC | 113 | NM_001012271 |
| ANXA1 | AATGCCTCACAGCTATCGT | TCCTCCACAAAGAGCCACC | 251 | NM_000700 |
| BMP4 | CCTGGGCACCTCATCACA | CATAGTTTGGCTGCTTCTC | 124 | NM_001202 |
| CCNB1 | CTAAGATTGGAGAGGTTGATGTC | GGTAATGTTGTAGAGTTGGTGTC | 177 | NM_031966 |
| CCND1 | GGTGGCAAGAGTGTGGAG | CCTGGAAGTCAACGGTAGC | 148 | NM_053056 |
| CDK1 | GGATGTGCTTATGCAGGATTCC | CATGTACTGACCAGGAGGGATAG | 100 | NM_001786 |
| CDKN2D | CCTCAACCGCTTCGGCAAGAC | ATCCAGTGCGGGCTGCGTCAT | 146 | NM_001800 |
| CREM | CTCTGATGTGCCTGGTGTT | AGCTGATTGTGCTGCGTAC | 262 | NM_181571 |
| CXCL3 | CGCCCAAACCGAAGTCATAG | GCTCCCCTTGTTCAGTATCTTTT | 109 | NM_002090 |
| EGR1 | CAGCAGCCTTCGCTAACC | CCACTGGGCAAGCGTAA | 184 | NM_001964 |
| F2RL1 | GGGTTTGCCAAGTAACGGC | GGGAACCAGATGACAGAGAGG | 123 | NM_005242 |
| HDAC9 | TTCCATGAACCTGCTAAGTC | ATGCTGCCTCCATACTGC | 174 | NM_058176 |
| IL15 | TCCTAAAACAGAAGCCAACT | TCCGGACTCAAGTGAAATA | 181 | NM_000585 |
| JUN | CGCCAAGAACTCGGACCTC | CCTCCTGCTCATCTGTCACG | 164 | NM_002228 |
| PPP2R1A | ACCGCATGACTACGCTCTT | TGGGCTTGACTTCACTCTG | 198 | NM_014225 |
| SFRP1 | AGTCGGACATCGGCCCGTAC | GTCTCGTGCTCCAGCAGGTT | 139 | NM_003012 |
| STIP1 | CCTGAGCGTGGGTAACATCG | AGCTGCTGCTTTTCGTGAAT | 202 | NM_006819 |
| TNFSF10 | CTGCCTGGCTGACTTACA | CGGAGTTGCCACTTGAC | 294 | NM_003810 |
| TOP2A | GGCACCAGCACATCAAAGG | TCAAACCATCCACCATAGAAGG | 319 | NM_001067 |
| ZFP36 | TCCCCATCTTCAATCGCATCT | TGAGGTCAGGGGAGTGGGTTA | 191 | NM_003407 |
